# Comparative Study of Voxel-Based Epileptic Foci Localization Accuracy between Statistical Parametric Mapping and Three-dimensional Stereotactic Surface Projection

**DOI:** 10.3389/fneur.2016.00164

**Published:** 2016-09-27

**Authors:** Kailiang Wang, Tinghong Liu, Xiaobin Zhao, XiaoTong Xia, Kai Zhang, Hui Qiao, Jianguo Zhang, Fangang Meng

**Affiliations:** ^1^Beijing Neurosurgical Institute, Capital Medical University, Beijing, China; ^2^Beijing Key Laboratory of Neurostimulation, Beijing, China; ^3^Department of Nuclear Medicine, Beijing Tiantan Hospital, Capital Medical University, Beijing, China; ^4^Department of Neurosurgery, Beijing Tiantan Hospital, Capital Medical University, Beijing, China

**Keywords:** epilepsy, statistical parametric mapping, ^18^F-FDG-PET, 3D-SSP, epileptic focus

## Abstract

**Introduction:**

Fluorine-18-fluorodeoxyglucose positron-emission tomography (^18^F-FDG-PET) is widely used to help localize the hypometabolic epileptogenic focus for presurgical evaluation of drug-refractory epilepsy patients. Two voxel-based brain mapping methods to interpret ^18^F-FDG-PET, statistical parametric mapping (SPM) and three-dimensional stereotactic surface projection (3D-SSP), improve the detection rate of seizure foci. This study aimed to compare the consistency of epileptic focus detection between SPM and 3D-SSP for ^18^F-FDG-PET brain mapping analysis.

**Methods:**

We retrospectively reviewed the clinical, electroecephalographic, and brain imaging results of 35 patients with refractory epilepsy. ^18^F-FDG-PET studies were revaluated by SPM, 3D-SSP, and visual assessment, and the results were compared to the magnetic resonance imaging (MRI) lesion location and to the presumed epileptogenic zone (PEZ) defined by video-electroencephalogram and other clinical data. A second consistency study compared PET analyses to histopathology and surgical outcomes in the 19 patients who underwent lesion resection surgery.

**Results:**

Of the 35 patients, consistency with the PEZ was 29/35 for SPM, 25/35 for 3D-SSP, 14/35 for visual assessment, and 10/35 for MRI. Concordance rates with the PEZ were significantly higher for SPM and 3D-SSP than for MRI (*P* < 0.05) and visual assessment (*P* < 0.05). Differences between SPM and 3D-SSP and between visual assessment and MRI were not significant. In the 19 surgical patients, concordance with histopathology/clinical outcome was 14/19 for SPM, 15/19 for 3D-SSP, 14/19 for visual assessment, and 9/19 for MRI (*P* > 0.05). A favorable Engel outcome (class I/II) was found in 16 of 19 cases (84%), and failure of seizure control was found in 3 of 19 patients (class III/IV).

**Conclusion:**

Voxel-based ^18^F-FDG-PET brain mapping analysis using SPM or 3D-SSP can improve the detection rate of the epileptic focus compared to visual assessment and MRI. Consistency with PEZ was similar between SPM and 3D-SSP; according to their own characteristics, 3D-SSP is recommended for primary evaluation due to greater efficiency and operability of the software, while SPM is recommended for high-accuracy localization of complex lesions. Therefore, joint application of both software packages may be the best solution for FDG-PET analysis of epileptic focus localization.

## Introduction

Up to 30% of individuals with epilepsy are estimated to be drug resistant, which was defined when epilepsy remains uncontrolled despite two adequate trials of antiepileptic drugs that were appropriate for the person’s disease (1), and these cases are challenging to manage (1–3). Fortunately, resective surgery can reduce or eliminate seizure occurrence for most cases and can be a powerful means of treating people with drug-resistant epilepsy (DRE). Seizures originate from one or more epileptogenic zones, and successful seizure control depends on their complete removal. Therefore, identification and precise localization of the epileptic focus is imperative for achieving optimal results following epilepsy surgery. Routine presurgical evaluation methods include semiology, computed tomography (CT), magnetic resonance imaging (MRI), electroencephalogram (EEG), and positron-emission tomography (PET) (4). Although intracranial EEG evaluation remains the gold standard for focus localization, surgical invasiveness and concomitant risks limit widespread use (5). Structural imaging by MRI can detect anatomic abnormalities (lesions) associated with epileptic focus, but many foci are MRI-negative. Alternatively, radionuclide imaging techniques, such as fluorine-18-fluorodeoxyglucose positron-emission tomography (^18^F-FDG-PET), identify functional brain abnormalities. Uptake of ^18^F-FDG measures regional cerebral glucose metabolism, which is often reduced in epileptic foci (6).

In clinical practice, a brain region with hypometabolism of ^18^F-FDG-PET is generally deemed to closely overlap with the epileptic foci during the interictal state (7). However, the accuracy of ^18^F-FDG-PET detection is still debated. For instance, Dellabadia et al. (8) reported a sensitivity of 86%, but Khan et al. (6) found only 59% sensitively, and Kumar et al. (9) reported 35–71% accuracy for identifying the resection site. These differences likely result from the methods used to analyze FDG-PET scans. Mainstream approaches to interpret PET results include subjective visual interpretation and objective methods, such as voxel-based statistical analysis with statistical parametric mapping (SPM) (10–14). Three-dimensional stereotactic surface projection (3D-SSP) with a different voxel-based computing paradigm, developed by Minoshima et al. (15), has been used to evaluate the spatial distribution of abnormal regional cerebral blood flow (rCBF) in SPECT and has also been applied to PET analysis, especially for Alzheimer’s disease lesions (characterizing AD with a ^18^F-FDG PET-specific pattern of prominent parietotemporal and posterior cingulate cortex hypometabolism using 3D-SSP.) (16), with encouraging results (17–19). A few previous epilepsy studies attempted to localize the epileptic focus using 3D-SSP. For instance, Singhal et al. (20) reported that 3D-SSP identified the hypometabolic zone corresponding to the surgical site in 36 of 40 cases (90%). However, no study has directly compared the accuracy of 3D-SSP to SPM for epileptic focus localization.

The purpose of our study was to compare 3D-SSP to SPM using the presumed epileptogenic zone (PEZ, defined by scalp video-EEG or invasive subdural EEG) as standards, which was verified by postoperative histology and outcomes in a proportion of patients.

## Materials and Methods

### Patients

From June 2014 to June 2015, we retrospectively reviewed 76 cases of medically intractable epilepsy evaluated for possible vagus nerve stimulation (VNS) therapy at our epilepsy center. The presurgical evaluation includes one or more of the following: seizure semiology, neurologic examination, brain MRI, interictal ^18^F-FDG-PET, and 24-h video scalp EEG monitoring. If the results were ambiguous or inconclusive, subdural EEG examination using strip and grid or depth electrodes was performed on the suspected area before surgery. Ultimately, the PEZ was defined by the scalp or subdural EEG results and clinical data (seizure semiology, neurologic examination). Patients were included in the study who met the following criteria: (a) patients had been diagnosed with intractable epilepsy by epileptologists according to the International League Against Epilepsy (ILAE) definition of DRE in 2009 (1); (b) patients should undergo sufficient preoperative assessment, including electrical physiological examination (scalp or subdural EEG), brain MRI, and interictal ^18^F-FDG-PET; then, 19 cases were excluded for whom the origin of epilepsy in MRI was completely concordant with video-EEG, and ^18^F-FDG-PET imaging was not conducted; and (c) the anatomical structure should be complete in the T1-weighted MRI, and not destructed by the suspect epileptic sites and surgical operation. Thirteen patients had significant space occupying lesions on MRI, such as glioma or dysembryoplastic neuroepithelial tumor (DNET), resulting in severe distortion of the surrounding tissues, and nine patients had undergone failed epilepsy surgery resulting in a clear brain tissue defect. Brain tissue defects and deformation are likely to cause errors in PET analysis (failure in PET data normalization), so these 22 patients were excluded. Finally, 35 patients (26 males, 9 females) aged 10–47 years (mean ± SD, 24 ± 11 years) were included in the study, as listed in Table 1.

**Table 1:** **The complete information of the all 35 patients, including imaging results, pathology results, and surgical outcome**.

No.	Sex	Age(y)	MRI	SPM-PET	3D-SSP	Visual-PET	PEZ	Surgery	Pathology	Engel
1	F	28	N	L T	L T	L T	L T	L ant_T resection	HS/m_T ulegyria	I
2	M	24	N	L T	L T	L T	L T	L ant_T resection	HS/m_T FCD	I
3	M	24	L T–P–O	L T–P–O (FDR)	L T–P–O	L T–P–O	L T–P–O	L T–P–O lesion resection	FCD	II
4	M	6	R T	R T	R T	R T	R T	R ant_T resection	HS/m_T sclerosis	I
5	M	44	N	R O	R T	R T	R T	R ant_T resection	HS/m_T sclerosis	I
6	M	24	N	R T–P–O	R T–P–O	R P	R T–P–O	R T–P–O lesion resection	FCD	I
7	M	31	L T	L F	L F	L T	L T	L ant_T resection	HS/m_T sclerosis	I
8	M	10	R T	R T/T–O/F (FDR)	R T/T–O	R T/P/O	R T	R post_T resection	encephalitis	IV
9	M	20	N	R T	R T	R T	R T	R ant_T resection	HS/m_T sclerosis	I
10	F	37	L T	L T	L T	L T	L T	L ant_T resection	HS/m_T FCD	I
11	M	38	N	R T	R T	N	R T	R ant_T resection	HS/m_T sclerosis	I
12	M	10	R insular	R P/insular	R T–P–O	R P/T	R insular	R insular lesion resection	FCD	III
13	M	18	R F	R F/T	R T	R F/T	R T/F	R ant_T resection; R F lesion resection	FCD	II
14	F	28	L T	L T	L T	L T	L T	L ant_T resection	HS/m_T sclerosis	I
15	M	38	L T	L T	L T	L/R T	L T	L ant_T resection	HS/m_T sclerosis	I
16	M	25	N	R T	R T	R T	R T	R ant_T resection	HS/m_T FCD	I
17	M	16	N	L T	L T	L T	L T	L ant_T resection	HS/m_T sclerosis	III
18	M	16	N	N	N	R T	R T	R ant_T resection	HS/m_T FCD	I
19	M	34	L T	L T	L T	N	L T	L ant_T resection	HS/m_T sclerosis	I
20	F	10	N	R F/P/SMA (FDR)	DIFF	L/R cerebellum	R SMA	VNS		
21	F	10	R T	L/R T (FDR)	L/R T	N	R/L T	VNS		
22	M	18	L T	R T–P–O	L F	R F	R T	VNS		
23	M	10	N	L/R cerebellum	L/R cerebellum	N	L/R cerebellum	VNS		
24	M	12	L/R F	L/R cerebellum	L/R cerebellum	N	L/R cerebellum	VNS		
25	M	18	N	DIFF	L/R cerebellum	N	L/R cerebellum	VNS		
26	M	29	N	DIFF	DIFF	N	DIFF	VNS		
27	F	11	N	L/R T	L/R T	N	L/R T	VNS		
28	M	21	N	L/R cerebellum	L/R cerebellum	R F	L/R cerebellum	VNS		
29	M	25	R T–P–O	R T–P–O	NONE	N	R primary motor	VNS		
31	F	28	N	DIFF	DIFF	N	DIFF	AED		
32	F	24	N	L T–P–O	N	N	L primary motor	AED		
32	M	22	N	L F/P	DIFF	N	L primary motor	AED		
33	M	47	N	DIFF	DIFF	N	DIFF	AED		
34	F	47	N	DIFF	DIFF	N	DIFF	AED		
35	M	42	N	R SMA/F	DIFF	N	R SMA	AED		

*y, year; F, female; M, male; L, left; R, right; T, temporal lobe; P, parietal lobe; F, frontal lobe; O, occipital lobe; SMA, supplementary motor area; ant_T, anterior temporal lobe; post_T, posterior temporal lobe; m_T, medial temporal lobe; T–P–O, junction of temporal–parietal–occipital lobe; T–O, junction of temporal–occipital lobe; T/P/O, all temporal lobe, parietal lobe, and occipital lobe. DIFF, diffuse into multi-lobes; N, normal; VNS, vagus nerve stimulation; AED, antiepileptic drugs; HS, hippocampal sclerosis; FCD, focal cortical dysplasia. FDR, FDR corrected*.

For SPM analysis, 25 healthy volunteers (20 men and 5 women, mean ± SD, 27 ± 5 years) who underwent physical examinations including whole-body PET at our PET Imaging Center served as controls. All PET scanning results in the control group were confirmed as normal. All controls were right-handed with no history of neurological, psychological, or severe medical illness requiring drugs known to affect brain ^18^F-FDG-PET studies. Informed written consent was obtained from all patients, and all procedures were approved by the Beijing Tiantan Hospital Ethics Committee.

Video-EEG monitoring and MRI imaging is detailed in the Supplementary Material and Table 1.

### ^18^F-FDG PET Imaging

All patients fasted for 4 h before ^18^F-FDG injection. Thirty-seven MBq of ^18^F-FDG per ten kilogram of body weight was injected intravenously in the awake and resting state, and PET image acquisition was started about 60 min after injection. The ^18^F-FDG-PET studies were performed using a GE Discovery 690 Medical system (Discovery ST, GE Healthcare, Waukesha, WI, USA). Computed attenuation correction was utilized to correct for attenuation of 511-keV photons. Emission images were reconstructed in a 192 × 192 × 47 matrix with a voxel size of 1.56 × 1.56 × 3.27 in the axial direction using the ordered subsets expectation maximization algorithm, with 5 iterations and 32 subsets, and corrected for attenuation using the CT transmission scan. To increase comfort and reduce fear and anxiety during the ^18^F-FDG uptake period, patients were not monitored by scalp EEG. However, no visible seizure events occurred. Sedation was adopted when necessary. Heart rate, blood pressure, and pulse oximetry were measured during PET.

### ^18^F-FDG PET Analysis

#### Visual Evaluation

Fluorine-18-fluorodeoxyglucose positron-emission tomography images were visually evaluated (visual-PET) by two investigators with expertise in reading PET images of the epileptic brain. The PET visual evaluators and data analysts of SPM and 3D-SSP were unaware of the findings of the video-EEG and MRI, and were blinded to the location of the PEZ. Hypometabolic brain areas were identified by consensus based on differences with regional uptake in the contralateral hemisphere.

#### SPM Analysis

Fluorine-18-fluorodeoxyglucose positron-emission tomography data were analyzed voxel-by-voxel using SPM8 (Wellcome Department of Cognitive Neurology, University College, London, UK) running on Matlab (Mathworks Inc., Sherborn, MA, USA) as described (21–23). First, PET images were co-registered with the SPM template T1-weighted MR reference to enable visualization and localization of activation loci. Co-registered PET images were then spatially normalized into a common Montreal Neurological Institute (MNI) atlas anatomical space following a 12-parameter affine transformation and non-linear transformations, yielding images composed of 2 mm × 2 mm × 2 mm voxels. Third, normalized images were smoothed with FWHM = 12 mm using Isotropic Gaussian Kernel to increase the signal to noise ratio.

Subsequently, preprocessed PET image values were corrected to a mean value of 50 mL/dL/min by “proportional scaling” to reduce individual variation. A mask with 0.8 intensity value was used to select only voxels with activity and to exclude extra cranial activities. A two-sample *t*-test, based on the specified contrasts (hypometabolism in patients), was applied between individual patient data and the control group and to best reduce the impact of age and gender, both factors were regressed out as covariates. The resulting *P* value and extended voxel size (*k*) were thresholded at two levels and three levels, respectively: *P* < 0.001 (matched with *k* > 50, 100, 200 voxel corrected, respectively) and *P* < 0.01 (matched with *k* > 50, 100, 200 voxel corrected, respectively). What’s more, we choose the little *P* value first, but the central hypometabolic regions should have been kept unchanged among at least two voxel sizes simultaneously.

#### 3D-SSP Analysis

NEUROSTAT 3D-SSP software (University of Michigan, Ann Arbor, MI, USA) conducts quantitative statistical analyses based on voxel computation (15). First, PET data were converted from the “dicom” format to a 32-bit REAL binary format supported by default in 3D-SSP. Second, we reduced the image size to 128 × 128 from 192 × 192. Individual PET images were analyzed automatically using 3D-SSP software. Patient PET data were compared to the age-matched healthy control group (embedded in the 3D-SSP), with voxel-by-voxel *Z*-score analysis after voxel normalization to values of four reference regions, global mean, pontine, cerebellar, and thalamus, where *Z* score = [(control mean) − (individual value)]/(control SD) as previously reported by Minoshima et al. (15). Deviations from normal values are expressed as a 3D-SSP *Z*-score map from the projection of lateral, anterior, posterior, superior, inferior, and medial views.

### Surgical Treatment and Follow-up

A subset of patients (19/35) with a definite epileptic zone (EZ) and no operative contraindications underwent resection surgery, including lobar or multi-lobar selective cortical resection. All resected brain tissues were sent to the neuropathology department for histopathologic analysis.

Postsurgical outcome was evaluated 6−14 months later (mean ± SD, 10 ± 3 months) according to Engel’s classification (24): class I (completely seizure-free, auras only, or atypical early postoperative seizures only), class II (≥90% seizure reduction or nocturnal seizures only), class III (≥50% seizure reduction), and class IV (<50% seizure reduction). Patients were then divided into two subgroups: favorable outcome (Engel class I/II) and non-favorable outcome (Engel class III/IV).

### Statistical Analysis

All neuroimaging and analysis results were revaluated by brain imaging experts and epilepsy specialists, and the location of the PEZ was determined. The following measures were introduced to classify the results. (i) Consistency study (CS) to see if the result was concordant with the PEZ; (ii) positive-consistency study (PCS) to see if the result was not concordant with the PEZ, but the EZ was in the same hemisphere as the PEZ; (iii) negative-consistency study (NCS) to see if the EZ location was in the contralateral hemisphere, or bilateral lesions were predicted when the PEZ was unilateral; and (iv) normal-study (NS) to see if imaging results were normal.

The proportions of each study classification for MRI, SPM-PET, 3D-SSP-PET, and visual-PET relative to PEZ are reported. Proportions were compared by the McNemar test with *P* < 0.05 considered significant. A kappa test was performed, and a *k-*statistic value was also calculated to measure the consistency rates among modalities. Statistical procedures were performed using SPSS (version 20.0; SPSS Inc., Chicago, IL, USA).

## Results

### Demographic Results

There were no significant differences between the demographic data of the two groups, including gender (*P* = 0.76) and age (*P* = 0.15). Chi-square test and Mann–Whitney *U*-test were performed for gender and age between epileptic patients and healthy controls, respectively.

### Seizure Localization by MRI, SPM-PET, 3D-SSP-PET, and Visual-PET

**Figure 1:**
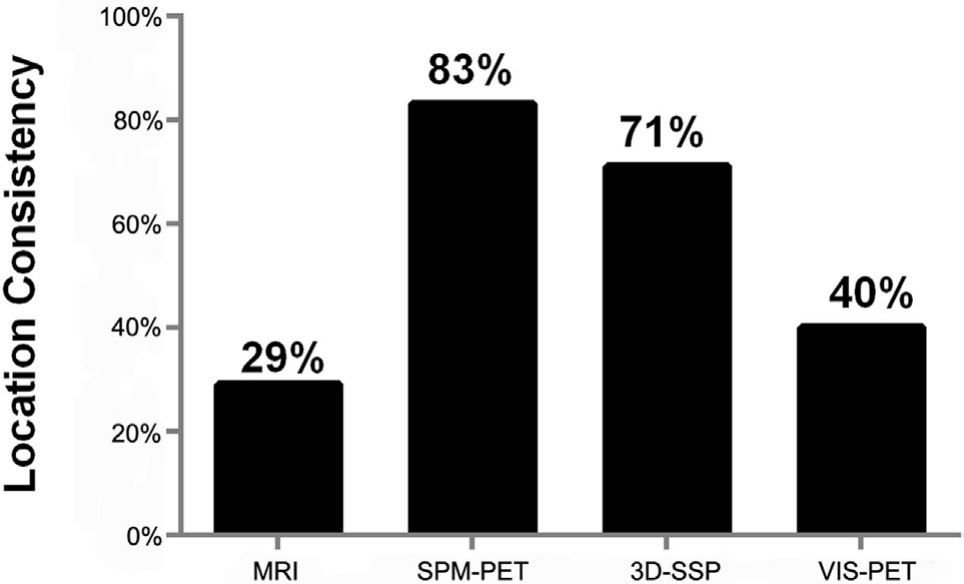
**Concordance of localization for each modality relative to the presumed epileptogenic zone (PEZ)**.

The epilepsy surgeon was able to define a clear PEZ in 31 of 35 patients (89%) by combining clinical data and video-EEG. In the remaining four patients, video-EEG showed dispersed activity and failed to detect single or specific seizures. All localization results were shown in Table 2 and Figure 1.

**Table 2:** **Classification of patients**.

Imaging modality	Normal-study (NS)	Consistency study (CS)	Positive-consistency study (PCS)	Negative-consistency study (NCS)
**(A) All 35 patients**				
MRI	21	10	2	2
VIS-PET	15	14	4	2
SPM-PET	1	29	5	0
3D-SSP-PET	3	25	3	4
**(B) 19 patients undergoing surgery**				
MRI	9	9	1	0
VIS-PET	2	14	2	1
SPM-PET	1	14	4	0
3D-SSP-PET	1	15	3	0

Magnetic resonance imaging findings were abnormal in 14 of 35 patients (40%). In 10 of 35 cases (29%), the MRI lesion was concordant with PEZ (CS), and in 2 of 35 cases (6%), it was not concordant but was in the same hemisphere (PCS). The other 2 cases were classified as NCS. No abnormalities were found in 21 of 35 (60%) MRI studies (NS).

According to visual evaluation, PET findings were abnormal in 20 of 35 patients (57%); 14 of 35 cases (40%) were classified as CS, 4 as PCS, and 2 as NCS. PET studies were classified as normal by visual evaluation in 15 of 35 cases (43%).

Statistical parametric mapping-PET findings were abnormal in 34 of 35 patients (97%), with 29 of 35 (83%) classified as CS and 5 as PCS. In one case (case 18), SPM-PET could not localize the epileptic focus. A more comprehensive analysis localized the epileptic foci to the right temporal lobe, according to the semiology and EEG results.

Three-dimensional stereotactic surface projection-PET results were abnormal in 32 of 35 patients (91%), with 25 of 35 cases (71%) classified as CS, 3 as PCS, and 4 as NCS. 3D-SSP-PET studies were classified as normal in 3 of 35 cases (9%).

**Figure 2:**
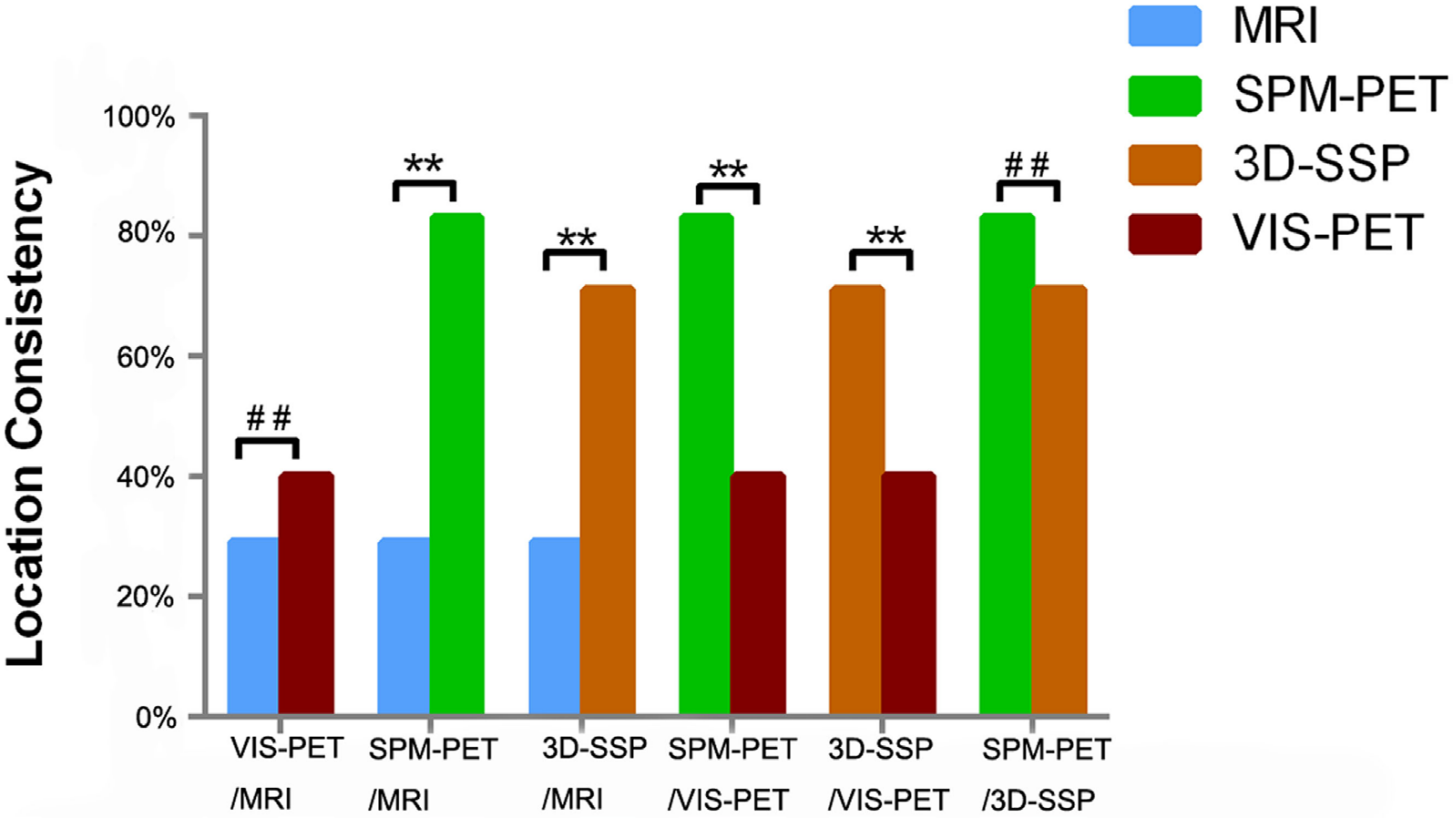
Comparison of location concordance relative to PEZ among analysis methods (**P < 0.05, ^##^*P* > 0.05).

**Figure 3:**
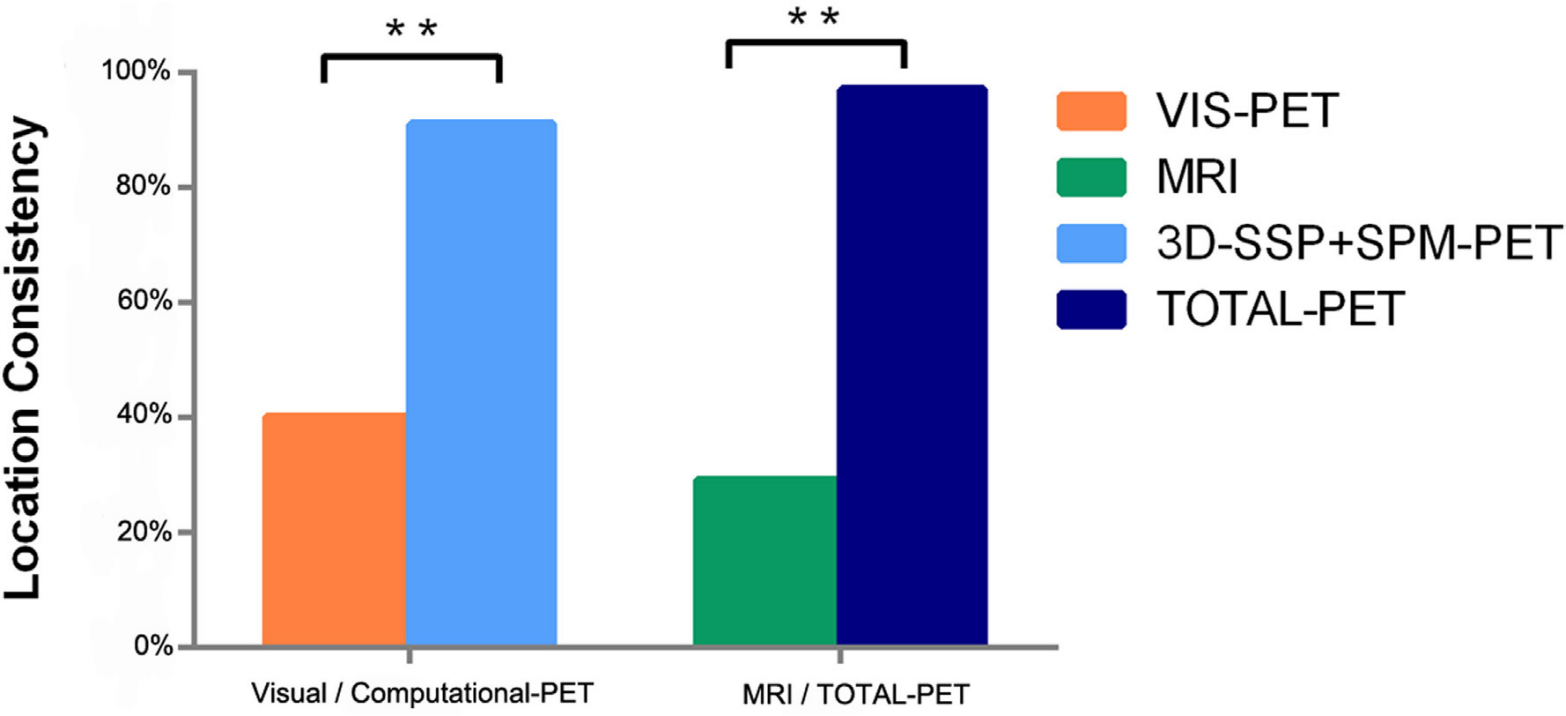
Comparison of computational methods (3D-SSP + SPM) to visual-PET interpretation and all PET methods to MRI (**P < 0.05).

The concordance rate with PEZ was significantly higher using computational analyses of PET compared to MRI (SPM-PET: 83 vs. 29%, *P* = 0.000, *k* = −0.115; 3D-SSP-PET: 71 vs. 29%, *P* = 0.001, *k* = −0.014), while the difference between visual-PET and MRI was not significant (40 vs. 29%, *P* = 0.388, *k* = 0.25), as shown in Figure 2. Combined SPM-PET and 3D-SSP-PET results were consistent with PEZ in 32 of 35 cases (91%), while combining computational analysis with visual-PET increased consistency with PEZ to 34 of 35 cases, significantly higher than for MRI (97 vs. 26%, *P* = 0.000, *k* = −0.058) (Figure 3). Indeed, MRI failed to reveal the epileptogenic lesion according to PEZ in 25 of 35 patients (71%). Within this NS group, SPM-PET successfully localized the PEZ-defined lesion in 22 of 25 cases (88%) and 3D-SSP-PET in 18 of 25 cases (72%), while visual-PET was consistent with PEZ in only 8 of these 25 cases (32%).

Computational PET analysis showed higher consistency with PEZ than visual-PET (SPM-PET: 83 vs. 40%, *P* = 0.003, *k* = −0.162; 3D-SSP: 71 vs. 40%, *P* = 0.013, *k* = 0.105), and visual-PET failed to localize the PEZ-defined epileptogenic lesion in 21 of 35 patients (60%). Within this NS group, SPM-PET was consistent with PEZ in 19 of 21 cases (90%) and 3D-SSP-PET in 14 of 21 cases (66%).

Though not significant, SPM-PET was more consistent with PEZ than 3D-SSP-PET (83 vs. 71%, *P* = 0.344, *k* = 0.205) with fair diagnostic consistency, as 3D-SSP-PET analysis failed to reveal an epileptogenic lesion that matched PEZ in 10 of 35 patients (29%). Within this NS group, SPM-PET was consistent with PEZ in 7 of 10 cases (70%).

### Surgical Results

A total of 19 patients underwent epileptic foci resection surgery, 18 single lobectomies, and 1 multi-lobar resection. Pathologic analysis of resected tissues revealed nine cases of hippocampal and medial temporal lobe sclerosis, four cases of simple focal cortical dysplasia (FCD), four cases of hippocampal sclerosis combined with medial temporal lobe FCD, one case of hippocampal sclerosis with medial temporal lobe ulegyria, and one case of encephalitis.

All surgical patients were followed up for at least 6 months (mean ± SD, 10 ± 3 months) after resection surgery. Engel’s class I was obtained in 14/19 cases, class II in 2/19, class III in 2/19, and class IV (i.e., unchanged) in 1 case. According to the binary classification of outcome, 16 of 19 patients (84%) had a favorable Engel outcome (class I/II), and 3 of 19 (16%) had an unfavorable Engel outcome (class III/IV).

**Figure 4:**
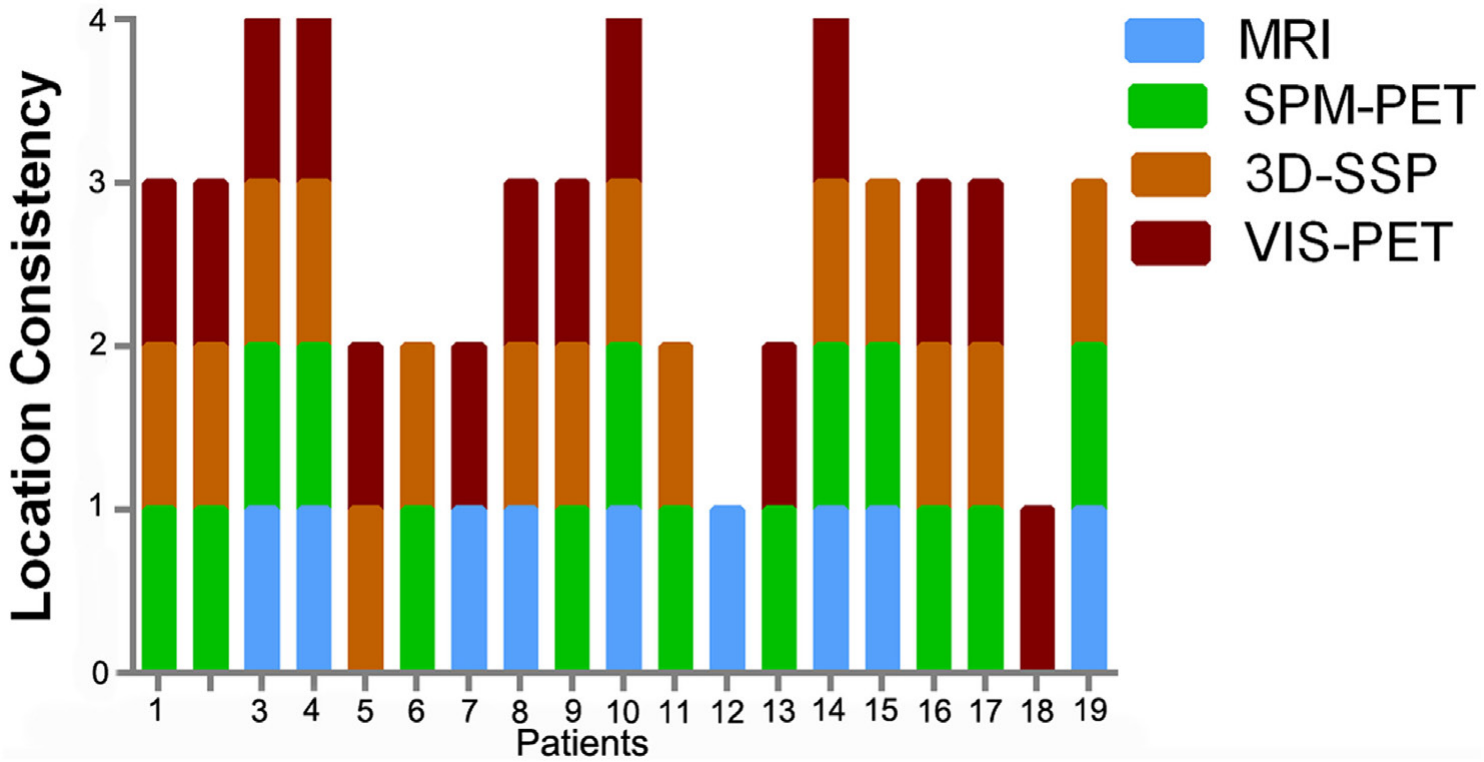
**Diagnostic accuracy of each imaging modality among the 19 surgical patients**. The columns represent the number of modalities consistent with PEZ for each case.

**Figure 5:**
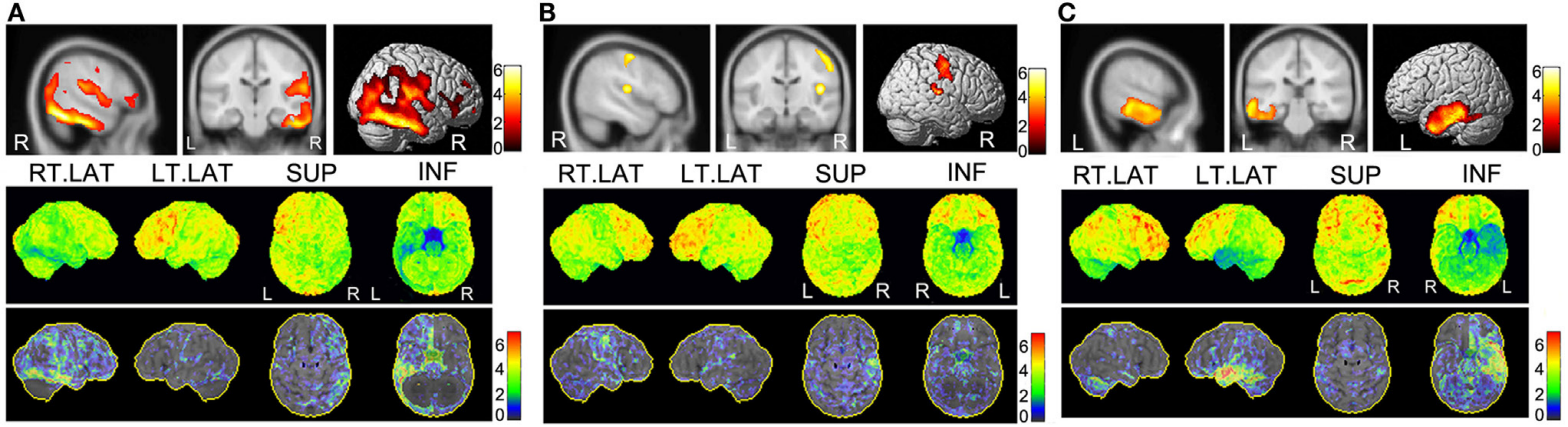
**PET imaging results of three cases with non-favorable outcome**. In each panel, the upper part is the SPM-PET result (*P* < 0.001, voxel size 200 corrected), and the lower part is the SD-SSP-PET result with *Z*-score. **(A)** Case 8 was diagnosed with encephalitis by postoperative pathological diagnosis. He received right posterior temporal lobe lesion resection with a very poor outcome (Engel IV). Both the SPM-PET and the 3D-SSP-PET result maps reveal a hypometabolic region in right temporal lobe. SPM-PET also shows another hypometabolic area in the right Sylvian fissure extending from the insula. However, the 3D-SSP-PET result map shows a diffuse hypometabolic distribution in the right parietal lobe and frontal lobe. **(B)** Case 12 underwent right insular lesion resection according to PEZ with a postoperative outcome of Engel III. The SPM-PET result map shows another epileptic locus in the right posterior central gyrus. In addition to a hypometabolic region in the right Sylvian fissure extending from the insula, the posterior central gyrus region shows a high *Z*-score in the 3D-SSP result. **(C)** Case 17 underwent left anterior temporal lobectomy with a non-favorable outcome (Engel III). SPM-PET and 3D-SSP-PET result maps show ideal consistency in the localization of a hypometabolic region in the left temporal lobe. Failure of seizure control may have resulted from removal of a region smaller than the real epileptic zone to avoid functional loss.

**Figure 6:**
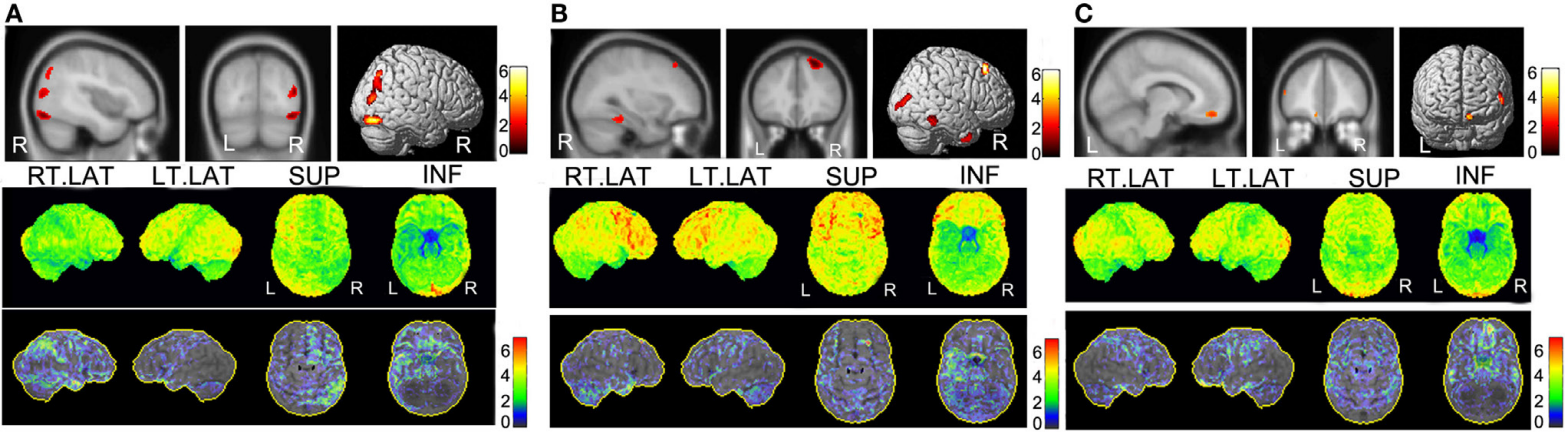
**PET imaging results of three cases with favorable outcome but were discordant between SPM and 3D-SSP**. In each panel, the upper part is the SPM-PET result (*P* < 0.001, voxel size 50 corrected), and the lower part is the SD-SSP-PET result with *Z*-score. **(A)** Case 5, the PEZ was localized at the right temporal lobe and underwent right anterior temporal lobe lesion resection. 3D-SSP showed a CS result with the PEZ of the right temporal lobe hypometabolism; however, SPM results were focused on the right occipital lobe hypometabolism. Considering the postsurgical pathology, the SPM result seemed untrustworthy. **(B)** Case 13, the right temporal and frontal lobes were considered to be PEZ. Overlapping hypometabolism regions were detected by SPM. In contrast, 3D-SSP only detected the epileptic focus in the right temporal lobe. Even though a very small metabolism was also found in the right frontal lobe by 3D-SSP, it is too small to account it as an epileptic focus, given the similar metabolism in the same position of the contralateral hemisphere. **(C)** Case 7, the PEZ was situated at the left temporal lobe and was confirmed by the postsurgical pathology. However, both 3D-SSP and SPM detected the hypometabolism of the left prefrontal lobe, not the temporal lobe.

Reasons for the unfavorable clinical outcomes were suggested by SPM and 3D-SSP analyses, detailed in Figures 4 and 5. In two cases (cases 8 and 12), EZ resection was incomplete as there was another epileptic foci aside from the PEZ. Although case 17 showed good consistency for all diagnostic methods, the resected region was likely smaller than the real EZ, so seizure control failed. In the favorable outcome group, there was discordance between SPM and 3D-SSP results in three cases (cases 5, 7, and 13) in Figure 6. More specifically, SPM showed PCS in case 5, 3D-SSP in case 13, and both in case 7.

## Discussion

Voxel-based analysis allows for objective identification of regional changes in brain structure and function, thereby improving diagnostic accuracy. SPM and 3D-SSP are both widely used for voxel-based analysis of brain PET (18, 21, 25–27). To the best of our knowledge, no study has directly compared 3D-SSP to SPM for the evaluation of ^18^F-FDG-PET in intractable epilepsy patients. We demonstrate that 3D-SSP is superior for localization compared to MRI and visual analysis of ^18^F-FDG-PET for localization, although not as accurate as SPM. Nonetheless, combined 3D-SSP, SPM, plus visual ^18^F-FDG-PET analysis was almost perfectly concordant with PEZ. Further, 3D-SSP and SPM revealed additional epileptogenic foci not found by PEZ that may be related to the poor outcome in several patients.

While not commonly applied for localization of epileptic foci, 3D-SSP has been shown to accurately distinguish the early stage of Alzheimer’s disease (mild cognitive impairment), frontotemporal dementia, and dementia with Lewy bodies from matched controls (16, 28, 29). In contrast to the SPM “two-sample *t*-test” calculation model, 3D-SSP uses a distinct *Z*-score calculation. Buchholz et al. (21) claimed that the extent of hypometabolism and peak *Z*-scores were influenced by the software calculation method rather than by different spatial normalization parameters. In the present comparative study, PEZ concordance was lower for 3D-SSP (25/35) than for SPM (29/35) in the entire cohort, but similar in the surgical patients (14/19 vs. 15/19). Excluding the 13 cases identified both by 3D-SSP and SPM, 3D-SSP but not SPM was concordant with PEZ in surgical cases 5 and 8, while SPM but not 3D-SSP was concordant with PEZ for surgical cases 12 and 13. Case 5 was HS/m_T sclerosis, and case 8 was encephalitis, while cases 12 and 13 were both FCD. 3D-SSP was reported to be less affected by the presence of atrophy than SPM in patients with AD (30), which is in agreement with our results as cases 5 and 8 showed more severe atrophy than cases 12 and 13. This indicates that the sensitivity of 3D-SSP depends on the histopathological type of epileptic foci. In some relatively deep medial cortical or subcortical regions, such as the medial supplementary motor area (SMA) and insula, 3D-SSP concordance with PEZ was substantially lower than SPM. Nonetheless, 3D-SSP was superior to visual analysis (71 vs. 40%) and MRI (71 vs. 26%). Further, SPM appeared to better localize multiple foci, while 3D-SSP yielded only a diffuse hypometabolic region. In the present study, 16 patients who received VNS or antiepileptic drugs are often with complex lesions, such as bilateral epileptic foci, multi-lobe sites, or some lesions related to brain functional areas. SPM localized the PEZ-defined lesions in 15 of 16 cases, compared to 10 of 16 cases by the 3D-SSP, and showed a higher consistency rate (94 vs. 67%, *P* = 0.125, *k* = −0.12). What’s more, most reported voxel-based ^18^F-FDG-PET epileptic focus studies adopted the SPM methods in the current paper and the published literatures we can retrieve, the 3D-SSP application in this field can overcome the selection bias.

The SPM-PET analytical method has been used for the presurgical localization of epileptic foci for over 15 years (31, 32), and its clinical utility has been confirmed in both adult and pediatric epilepsy patients (9). Like the general significance test model, its accuracy for epileptic foci localization depends on the statistical threshold and the voxel size, but the optimal tradeoff between these two parameters is still undefined. The SPM software default statistical threshold is *P* < 0.001, but there is no recommended cluster extent threshold. To date, most researchers have adopted thresholds of *P* = 0.01, 0.001, or 0.0001, false discovery rate (FDR) corrected *P* = 0.05, or family-wise (FWE) corrected *P* = 0.01, and most studies have set the voxel size to >50, 100, or 200 (9, 33, 34). Although some researchers have found that all parameters perform similarly well for the detection of epileptic foci (9), our experience is that the size of the lesion correlates with the extent of resection and functional loss. Here, we introduce a process to optimize SPM analysis. The most important factor is the statistical threshold of *P*. In our routine work, we use a threshold gradient of *P* = 0.001, 0.01, and 0.05. Second, an ascending voxel size gradient (50, 100, and 200) is recommended. The optimal result is chosen based on maintaining an unchanging central hypometabolic region among at least two voxel sizes under one or two conditions of *P* values simultaneously. With this method, we can detect hypometabolic brain regions that would be missed if we selected an inappropriate control group, thereby increasing the detection rate of negative SPM results. Here, we attach little importance to the statistical threshold correction by FDR or FWE for EZ localization in individual patients, and agree with Kumar et al. who found that uncorrected statistical thresholds performed well in obtaining clinically useful results for lobar localization. Alternatively, the stricter corrected threshold, though highly specific, yielded unacceptably low sensitivity.

Following this method, SPM-PET analysis presented the highest concordant rate with the PEZ and detected single epileptic foci missed by 3D-SSP, visual-PET, and MRI, and even multiple epileptic foci not detected by video-EEG and other clinical data. Thus, SPM-PET evaluation can define the optimum minimum epileptic focus for “subtotal” resection. In contrast, 3D-SSP defined a more diffuse EZ within a relatively larger brain volume. Seizures arising from medial (parasagittal) cortical foci are often challenging to identify for surgical resection. Thus, we confirm the findings of Kumar et al. that SPM appears to be a particularly useful approach for detecting medially located seizure foci.

Despite the lower concordance with PEZ compared to SPM, 3D-SPP was still much more accurate than visual-PET and MRI. Moreover, the 3D-SPP analytic procedure employs a more user-friendly graphical interface than that used for SPM, and the program produces color scaled *Z*-score maps from which the number of SDs from the mean of normal subjects is easily read, especially for the novice, allowing them to achieve the same detection accuracy as expert brain FDG-PET readers. In addition, the 3D-SPP program has built-in age-matched and normal databases that may obviate the need for a normal control group. However, although a *Z*-score of 1.5 or greater is regarded as indicative of pathology in AD diagnosis, the best threshold *Z*-score for epilepsy has not been established. The 3D-SSP shows results relative to four specific regions (GLB-global brain, thalamus, cerebellum, and pons), and these four comparators may reduce errors arising from a single comparator. In addition, 3D-SSP has greater software efficiency and operability, requires no previous programing experience, which was indispensable for learning SPM, and according to our experience, beginners can master 3D-SSP within a day, whereas 2 weeks or more time would be needed for learning SPM. Furthermore, length of time per patient with each analysis was shorter for 3D-SSP than for SPM, with about 2 min for 3D-SSP and 10 min or much longer for the latter. We strongly recommend 3D-SSP for routine epileptic localization due to the fast analysis procedure and relatively high accuracy.

Accurate localization of epileptic foci is critical for prognosis and to weighing surgical risks against postoperative benefits (4, 35). While both 3D-SSP- and SPM-PET analyses are more concordant with PEZ, MRI reveals the distinct anatomical structure around the focus that is essential for resection surgery, and EEG is still the gold standard for diagnosis of epilepsy. Thus, integration of multimodal neuroimaging, electrophysiology, and semiology provides the most reliable presurgical evaluation.

This study has several limitations. First, our patient selection criteria may have led to an underestimation of MRI sensitivity, as cases requiring only MRI or with obvious MRI deformation were excluded. Since more concerned with PET results, here, we introduced only MRI visual analysis results, and quantitative analysis of structural MRI was not used. Therefore, it is expected decreased MRI accuracy. Second, the standard for EZ localization (PEZ) was defined by video-EEG, clinical evaluation, and neuropsychological data. Therefore, PEZ is not absolutely definitive, and in some cases, PEZ and EZ did not match, especially in the VNS and AEDS groups in which the PEZ was not consistent with pathology findings. However, in the surgical subset (19/35), the PEZ-defined region was verified by histopathology. Third, the present PET studies were limited to FDG. Other PET tracers, such as tracers labeled with ^11^C (36), may show different concordance rates among PEZ, MRI, SPM, 3D-SSP, and visual-PET.

## Conclusion

Three-dimensional stereotactic surface projection and SPM ^18^F-FDG-PET assessment provided important complementary presurgical information that was concordant with PEZ in 32/35 of our samples. Computational PET analysis was particularly useful in those cases in which MRI and visual-PET did not identify an epileptogenic focus. SPM concordance was higher than 3D-SSP concordance. We believe that a correct interpretation of hypometabolic epileptic foci should integrate both SPM and 3D-SSP ^18^F-FDG-PET evaluation; 3D-SSP is recommended for primary evaluation due to software efficiency and operability, while SPM is recommended for deeper foci and more accurate localization. These techniques may extend the possibility of surgical treatment to patients previously considered inoperable.

## Author Contributions

KW: study concept and design, analysis of data, complete the manuscript. TL, XZ, and XX: acquisition and analysis of data. KZ and HQ: clinical data analysis. JZ: perform the resection surgery. FM: study concept and design, critical revision of manuscript for intellectual content.

## Conflict of Interest Statement

The authors declare that the research was conducted in the absence of any commercial or financial relationships that could be construed as a potential conflict of interest.
