# Supplementary material for: Comparative Study of Voxel-Based Epileptic Foci Localization Accuracy between Statistical Parametric Mapping and Three-dimensional Stereotactic Surface Projection
Source: Front Neurol. 2016 Sep 27;7:164. doi: 10.3389/fneur.2016.00164 (PMC5037321; doi:10.3389/fneur.2016.00164)
Supplement: Supplementary file 1 [file presentation_1.pdf]

Supplementary Material

Comparative study of epileptic foci localization accuracy between statistical parametric mapping (SPM) and Three-dimensional stereotactic surface projection (3D-SSP)

Kailiang Wang, Tinghong Liu, Xiaobin Zhao, XiaoTong Xia MD, Kai Zhang, Hui Qiao phD, Jianguo Zhang, Fangang Meng*.

* Correspondence: Fangang Meng: fgmeng@ccmu.edu.cn

Video-EEG Monitoring and MRI Imaging

All patients were examined by video-EEG monitoring in the epilepsy unit for at least 24 h, and AEDs were reduced or stopped when necessary to facilitate seizure occurrence. The EEG was recorded continuously from 19 active scalp electrodes or subdural strips and grids or depth electrodes using the NicoletOne EEG Monitoring system (Natus Medical Inc.) and interpreted by two epileptologists with extensive experience in EEG reading and epilepsy localization. In the current study, aside from 24h-video-scalp EEG monitoring which all patients have taken, 6 patients underwent the invasive subdural EEG examination for their localization of complex lesions.

MRI was performed using a 3.0-T unit (Genesis Signa) with a specific epilepsy protocol including T1-weighted, T2-weighted, and FLAIR sequences. Sedation was used in selected cases. All MRI studies were interpreted visually by a neuroradiologist and two epileptologists.
